# A Case of Gastric Neuroendocrine Carcinoma with Disseminated Recurrence 14 Years after Initial Surgery

**DOI:** 10.70352/scrj.cr.25-0285

**Published:** 2025-08-19

**Authors:** Takara Kinjo, Keishi Okubo, Masahiro Hamanoue, Miki Murakami, Takao Ohtsuka, Sonshin Takao

**Affiliations:** 1 Department of Surgery, Tanegashima Medical Center, Nishinoomote, Kagoshima, Japan; 2 Department of Pathology, Graduate School of Medical and Dental Sciences, Kagoshima University, Kagoshima, Kagoshima, Japan; 3 Department of Digestive Surgery, Graduate School of Medical and Dental Science, Kagoshima University, Kagoshima, Kagoshima, Japan

**Keywords:** gastric neuroendocrine carcinoma, disseminated recurrence, postoperative recurrence

## Abstract

**INTRODUCTION:**

Gastric neuroendocrine carcinoma (NEC) is a rare disease among gastric cancers, accounting for only 0.1%–0.6% of all cases. This disease is known to have a poor prognosis and a higher risk of recurrence compared to conventional gastric adenocarcinoma.

**CASE PRESENTATION:**

At the age of 44, a 60-year-old female underwent a laparoscopic-assisted proximal gastrectomy for gastric cancer at a previous hospital. Neuroendocrine carcinoma was diagnosed following a postoperative pathological examination based on histological findings and immunostaining results. The patient was followed up without any recurrences. After 14 years, a follow-up contrast-enhanced CT revealed a 9-mm mass on the greater curvature side of the gastric antrum, which was suspected to be lymph node swelling at the previous hospital. After 8 months, she came to our hospital with abdominal discomfort and distention. The CT scan revealed a 55-mm mass, indicating an increase in the previously mentioned mass. At our hospital, the patient underwent open tumor resection. The pathological findings revealed a recurrence of gastric NEC. The patient has been recurrence-free for 6 months following surgery.

**CONCLUSIONS:**

We present a case of gastric NEC with disseminated recurrence. To our knowledge, this is the first report of a disseminated case in which a recurrent lesion caused by omental dissemination grew in size and infiltrated a portion of the gastric serosa approximately 14 years after the initial surgery.

## Abbreviations

CSCs
: cancer stem cells
DTCs
: disseminated tumor cells
GIST
: gastrointestinal stromal tumor
HPF
: high-power field
NEC
: neuroendocrine carcinoma
NENs
: neuroendocrine neoplasms
WHO
: World Health Organization
UICC
: Union for International Cancer Control

## INTRODUCTION

Gastric neuroendocrine carcinoma (NEC) is a rare disease, accounting for only 0.1%–0.6% of all gastric malignancies.^1)^ It is thought to have an extremely poor prognosis, as the 5-year survival rate is reported to be 26.7%–44.7%,^2,3)^ with a median overall survival of 7 months.^4)^ Compared to gastric adenocarcinoma, gastric NEC has a higher risk of recurrence, with most postoperative recurrences occurring within 1–2 years, and recurrences lasting more than 5 years being rare.^5)^ We present a case of recurrence of gastric NEC more than 10 years after the initial surgery.

## CASE PRESENTATION

**Fig. 1:**
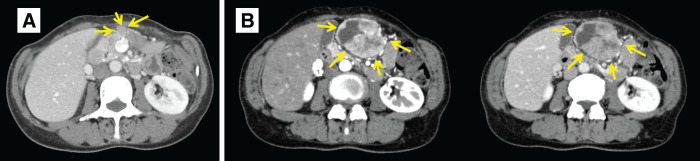
Preoperative CT images. (**A**) CT scan conducted at a previous hospital. It showed a 9-mm mass on the greater curvature side of the gastric antrum (arrows). (**B**) CT scan conducted at our hospital. A 55-mm hypervascular mass with internal necrosis and a clear border was observed at the sub-umbilical level (arrows). The tumor demonstrated early enhancement in the arterial phase (left image), with a decrease in the contrast effect in the late phase (right image).

**Fig. 2:**
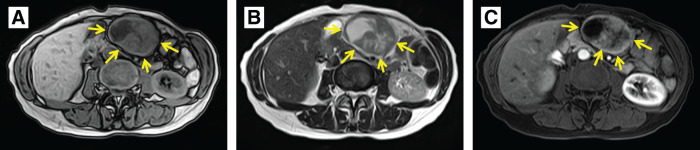
Preoperative MRI. (**A**) T1-weighted image. The tumor displayed low signal intensity (arrows). (**B**) T2-weighted image. The tumor showed a mixture of low and remarkably high signal intensities (arrows). (**C**) Contrast imaging demonstrated an early enhancement effect (arrows).

**Fig. 3:**
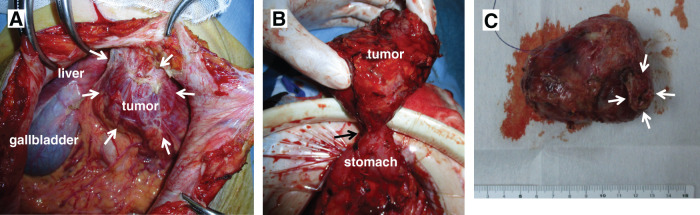
Surgical findings. (**A**) The tumor (arrows) was in contact with the abdominal wall, the omentum, and the stomach wall. (**B**) The tumor was in contact with the stomach wall (arrow); a combined resection of the stomach wall was performed. (**C**) The tumor was oval, measuring 8.0 × 5.5 × 3.5 cm. No notable findings were observed in the resected gastric mucosa (arrows).

**Fig. 4:**
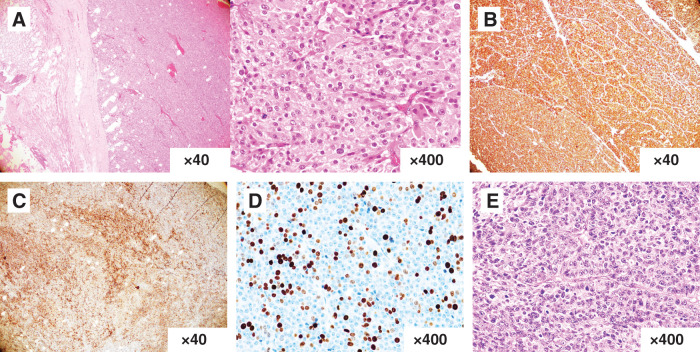
Pathological findings. (**A**) Hematoxylin–eosin staining. The tumor consisted of atypical cells with rounded, enlarged nuclei undergoing proliferation, with a mitotic index of more than 40/10 HPF. (**B**, **C**) Immunostaining. The tumor demonstrated positive staining for chromogranin A (**B**) and synaptophysin (**C**). (**D**) The Ki-67 labeling index was more than 60%. (**E**) Hematoxylin–eosin staining. The gastric cancer from 14 years ago appeared similar to the current tumorous lesion. HPF, high-power field

At the age of 44, a 60-year-old female underwent a laparoscopic-assisted proximal gastrectomy for gastric cancer at a previous hospital. Neuroendocrine carcinoma was diagnosed following a postoperative pathological examination based on histological findings and immunostaining results. The tumor measured 20 × 25 × 10 mm and was classified as type 0−IIa + IIc. It was located below the fenestra, on the lesser curvature side. The depth of invasion was T1 (SM2), with 2 metastases discovered in the No. 3 lymph nodes adhering to the serosal surface under the tumor, leading to a diagnosis of N1. Immunohistochemically, the tumor cells were positive for chromogranin A and synaptophysin. According to the Union for International Cancer Control (UICC) TNM classification, the diagnosis was pT1N1M0, pStage IB. The patient underwent 6 months of S-1 monotherapy. At her request, she had contrast-enhanced CT once a year and esophagogastroduodenoscopy every 2–3 years, even more than 5 years after the initial surgery, and was followed up without recurrence for 14 years. However, 14 years after the initial surgery, a follow-up CT showed a 9-mm mass on the greater curvature side of the gastric antrum, which was suspected to be lymph node swelling at the previous hospital (**Fig. 1A**). Esophagogastroduodenoscopy was performed 8 days after CT imaging, but no abnormal findings were noted. After 8 months, she complained of abdominal discomfort and distension, and visited our hospital for the first time. A contrast-enhanced CT scan revealed a 55-mm mass, indicating an increase in the previously mentioned mass (**Fig. 1B**). MRI showed a well-defined mass with low signal intensity on T1-weighted images (**Fig. 2A**) and a combination of pale high signal intensity and remarkable high signal intensity on T2-weighted images (**Fig. 2B**). Contrast-enhanced MRI showed an early contrast effect (**Fig. 2C**). Esophagogastroduodenoscopy did not reveal any obvious tumor. We suspected a gastrointestinal stromal tumor (GIST), leiomyosarcoma, lymph node metastasis, or peritoneal dissemination of gastric cancer. Consequently, we performed open tumor resection. Surgical investigations revealed that the tumor was in contact with the abdominal wall, the omentum, and the stomach wall (**Fig. 3A**). There were no obvious liver metastases or other peritoneal dissemination. The tumor was located in the omentum and was in contact with the stomach wall, so we performed a combined resection of the stomach wall (**Fig. 3B** and **3C**). Pathological findings indicated a recurrence of gastric NEC. Histologically, the tumor consisted of atypical cells with rounded, enlarged nuclei undergoing proliferation, and the mitotic index was more than 40/10 high-power field (HPF) (**Fig. 4A**). No adenocarcinoma component was observed. There were no abnormal findings in the resected gastric mucosa. Immunohistochemically, the neuroendocrine component revealed positive staining for chromogranin A and synaptophysin (**Fig. 4B** and **4C**), and negative staining for somatostatin receptor 2. The Ki-67 labeling index was more than 60% (**Fig. 4D**). The histopathological examination of the gastric cancer from 14 years ago was similar to the current tumorous lesion (**Fig. 4E**). Additionally, immunostaining showed positivity for synaptophysin and chromogranin A, indicating that it was a recurrent nodule of gastric NEC. Although preoperative CT suggested possible lymph node swelling, pathological examination did not reveal findings indicative of lymph node metastasis. Postoperatively, she has been treated with etoposide and cisplatin chemotherapy and remains well 6 months after surgery. We are planning to perform a total of 4–6 cycles of postoperative adjuvant chemotherapy for this patient.

## DISCUSSION

This case involves a patient with gastric NEC experiencing peritoneal dissemination recurrence approximately 14 years after the initial surgery. To our knowledge, this is the first reported case of such dissemination recurrence. Neuroendocrine neoplasms (NENs) of the pancreas and gastrointestinal tract are relatively rare tumors, with an annual incidence of 3.51 new cases per 100000 population.^6)^ In the World Health Organization (WHO) classification revised in 2019, NENs are broadly categorized into neuroendocrine tumors (NETs), which are highly differentiated, and NECs, which are poorly differentiated. Additionally, mixed neuroendocrine–non-NENs, where neuroendocrine and non-neuroendocrine tumor components coexist, have been proposed.^7)^ Gastric NEC is reported to progress more rapidly than conventional adenocarcinoma, with 85% being discovered as advanced cancer.^8)^ It is also prone to distant metastasis, particularly liver metastasis, which occurs at a high frequency of 52.5%.^2)^ For diagnosing gastrointestinal NENs, including those in the stomach, endoscopic examination, endoscopic biopsy, and endoscopic ultrasound-guided fine-needle aspiration (EUS-FNA) are generally recommended. However, in this case, no significant findings were observed endoscopically, and the preoperative CT scan did not indicate continuity with the stomach. As a result, EUS-FNA was not conducted. Characteristic imaging findings of pancreatic and gastrointestinal NENs include many being homogeneous hypervascular tumors, which are strongly enhanced in the arterial phase of dynamic CT scans.^9)^ MRI typically shows low signal intensity on T1-weighted images and high signal intensity on T2-weighted images.^9)^ Upon reviewing the imaging studies in this case, the contrast-enhanced CT and MRI revealed early strong enhancement in the arterial phase, and MRI revealed low signal intensity on T1-weighted images and heterogeneous high signal intensity on T2-weighted images, showing the characteristic imaging findings of NENs.

In this case, immunostaining showed positivity for chromogranin A, synaptophysin, and Ki-67. Similarly, 14 years ago, the tumor cells exhibited positive staining for chromogranin A and synaptophysin. Furthermore, the tissue morphology resembled that of the gastric cancer observed 14 years ago, leading to a diagnosis of NEC recurrence after surgery. Regarding the recurrence pattern, the pathological examination did not indicate lymph node metastasis, and preoperative CT ruled out continuity with the stomach. Therefore, it was considered that the recurrent lesion, resulting from omental dissemination, increased in size and infiltrated a part of the gastric serosa.

Gastric NEC has a higher risk of recurrence compared to conventional gastric adenocarcinoma, with postoperative recurrence rates ranging from 30.8% to 47.4%.^10,11)^ Most recurrences occur within 1–2 years, and recurrences beyond 5 years are rare.^5)^ Lin et al.^10)^ reported that among 427 cases of gastric NEC that underwent curative resection, liver metastasis was the most prevalent form of postoperative recurrence at 14.3%, while peritoneal disseminated recurrence was observed in 4.7% of cases. Furthermore, there are no other case reports that presented late recurrence of NEC after 14 years. Breast cancer is generally known for its late recurrence. Two hypotheses have been proposed to explain the mechanism behind this late recurrence slow-growing theory, which suggests that tumors with low proliferative activity gradually increase in size over an extended period until they become clinically apparent^12)^; and the tumor dormancy theory, which proposes that cancer cells remain dormant for a certain time before resuming proliferation and manifesting as recurrent lesions.^13)^ If this case is to be interpreted under one of these theories, the slow-growing theory seems inadequate, considering the rapid progression in a short period. Furthermore, studies analyzing disseminated tumor cells (DTCs) suggest that cancer metastasis and dissemination occur during the early phase of tumor development.^14,15)^ After leaving the primary cancer, DTCs may arrive at metastatic sites and remain dormant.^14,15)^ It is considered that these dormant DTCs become activated later due to various factors, such as immune components and systemic inflammatory reactions.^14,15)^ This observation appears to support the tumor dormancy theory. In recent years, it is considered that cancer stem cells (CSCs) have been implicated in recurrence, metastasis, and resistance to treatment in malignant tumors.^15,16)^ In particular, the dormancy and reactivation of CSCs are considered to be involved in late recurrence.^15,16)^ Referring to these theories, the mechanism of late recurrence in this case may be interpreted as follows: these cells that had disseminated to the omentum before the initial surgery remained dormant and were reactivated by certain triggers after 14 years, leading to rapid tumor growth.

There is not yet a full consensus on the standard treatment for pancreatic and gastrointestinal NENs, but surgical resection is the only approach expected to achieve a complete cure. If there is no unresectable local invasion or distant metastasis, surgical resection is the primary choice.^17)^ For NEC, if resection is feasible, the removal of both the primary and metastatic lesions is considered. However, even for local lesions, resection alone often results in poor treatment outcomes.^18)^ Therefore, surgery should be performed as part of a multidisciplinary treatment approach, incorporating chemotherapy and radiation therapy. Four to six cycles of chemotherapy with platinum-based agents are recommended for postoperative adjuvant chemotherapy of NEC.^19)^ Gastric NEC is a disease that should be considered for its potential to recur regardless of the stage or postoperative period. Even in cases where curative resection has been conducted, long-term follow-up is considered necessary.

## CONCLUSIONS

We report a case of gastric NEC with disseminated recurrence approximately 14 years after the initial surgery. Gastric NEC has a very poor prognosis, and long-term follow-up is essential due to the possibility of recurrence.

## DECLARATIONS

### Funding

None of the authors received financial support for this study.

### Authors’ contributions

TK and KO drafted the manuscript.

TK, KO, and MH collected data and performed the operation.

MM contributed to the pathological analysis.

TO and ST supervised the writing of the manuscript.

All authors discussed the manuscript content.

All authors read and approved the final manuscript.

### Availability of data and materials

The datasets supporting the conclusions of this article are included within the article.

### Ethics approval and consent to participate

This case report does not require ethics approval. Informed consent to participate in this study was obtained from the patient.

### Consent for publication

The patient consented to the publication of this case report and images.

### Competing interests

The authors declare that they have no competing interests.

### Use of artificial intelligence (AI) tools

We used the AI tool “Copilot” for spelling and grammar checking to improve the readability of the manuscript.
